# LASER: Large genome ASsembly EvaluatoR

**DOI:** 10.1186/s13104-015-1682-y

**Published:** 2015-11-24

**Authors:** Nilesh Khiste, Lucian Ilie

**Affiliations:** Department of Computer Science, University of Western Ontario, London, ON N6A 5B7 Canada

**Keywords:** Bioinformatics, DNA sequencing, Genome assembly, Assembly evaluation

## Abstract

**Background:**

Genome assembly is a fundamental problem with multiple applications. Current technological limitations do not allow assembling of entire genomes and many programs have been designed to produce longer and more reliable contigs. Assessing the quality of these assemblies and comparing those produced by different tools is essential in choosing the best ones. The QUAST program has become the current state-of-the-art in quality assessment of genome assemblies. The only drawback of QUAST is high time and memory usage for large genomes, e.g., over 4 days and 120 GB of RAM for a single human genome assembly.

**Results:**

We introduce LASER, a new tool for assembly evaluation that improves greatly the speed and memory requirements of QUAST. For a human genome assembly, LASER is 5.6 times faster than QUAST while using only half the memory; one human genome assembly is evaluated in 17 hours instead of 4 days. The code of LASER is based on that of QUAST and therefore inherits all its features.

**Conclusions:**

Genome assembly evaluation is an essential step in assessing the quality of an assembly that is too often done improperly, in part due to significant resource consumption. With the introduction of LASER, proper evaluation can be performed efficiently.

## Background

The current sequencing technologies produce short pieces of DNA, called reads, that need to be assembled together to reconstruct the original genome. Usually, whole genomes cannot be produced and instead the assembling programs produce longer DNA pieces, called contigs. High quality assemblies require longer and more accurate contigs. Genome assembly is a difficult problem that is far from being solved. A multitude of assemblers have been designed, see, e.g., [1–11].

Comparing the quality of two assemblies is already nontrivial; one may have longer contigs while the other may have fewer misassembles. Given the large number of tools available, choosing the best one for, say, building a new pipeline, becomes a difficult problem. Evaluating the assembly quality for an assembler during the designing stage is essential as well. Therefore, fast and effective evaluation of genome assembly quality is of crucial importance and a number of solutions have been proposed [12–17]. The most comprehensive evaluation is currently provided by the QUAST program [17]. QUAST quickly became the current state-of-the-art in assembly evaluation. Its thorough evaluation, new metrics, and useful visualizations made it achieve widespread use. Its only drawback is the high time and memory usage for large genome assemblies. In most cases, it requires over 4 days and 120 GB of RAM to assess the quality of a single human genome assembly.

To remedy this problem we have designed LASER: Large genome ASsembly EvaluatoR. LASER’s code is based on that of QUAST, inheriting all its features and advantages. We describe below the essential improvements implemented in LASER and compare its performance with that of QUAST on several human datasets.

## Methods

The most time consuming stage of QUAST is, by far, the maximal exact match (MEM) computation step of the alignment process, performed using the NUCmer aligner from MUMmer v3.23 [18]. Our recent E-MEM tool [19] clearly outperforms not only MUMmer but also the currently best tools for MEM computation in large genomes: [20–24]. It was therefore a natural choice for replacing MUMmer.

Besides using E-MEM, we performed a number of other improvements as well. A large number of redundant string copy operations on large strings in the ‘show-snp’ utility program of the MUMmer toolkit have been avoided. The memory and performance of Python code was improved by replacing class objects with tuples.

The rest of QUAST code has been reused in LASER. MUMmer and GlimmerHMM [25] are open source and the authors of GeneMarkS [26] have kindly allowed us to use their code in LASER.

## Results

As mentioned before, all features of QUAST have been preserved and LASER has been designed to be used exactly the same way as QUAST. That is, LASER produces exactly the same output. The advantage of LASER consists of greatly increased speed and reduced memory usage. To prove these claims, we have compared LASER and QUAST on several datasets, presented in Table 1. As we are interested in improvement when it really matters, that is, for large genomes, all datasets are human. They were all produced by Illumina HiSeq2000 machines. All datasets were assembled using SOAPdenovo2 [6]. We used SOAPdenovo2 because of its good speed. The k-mer size producing the best assembly (as indicated by the aligned N50 size) was used. This was $k=65$ for $\mathrm H_1$ and $k=71$ for the other datasets. The assemblies are available for download from the website of LASER.

All tests were performed on a DELL PowerEdge R620 computer with 12 cores Intel Xeon at 2.0'GHz and 256 GB of RAM, running Linux Red Hat, CentOS 6.3.

**Table 1:** The datasets used for comparison; accession numbers are included for the datasets and for the corresponding reference genomes

Dataset	Organism	Accession number	Read length	Number of reads	Total bp	Depth of coverage	Reference genome	Genome length
$\mathrm H_1$	*Homo sapiens*	SRR1302280	101	1,287,175,558	130,004,731,358	41	Build 38	3,209,286,105
$\mathrm H_2$	*Homo sapiens*	ERR194146	101	1,626,361,156	164,262,476,756	51	Build 38	3,209,286,105
$\mathrm H_3$	*Homo sapiens*	ERR194147	101	1,574,530,218	159,027,552,018	50	Build 38	3,209,286,105
$\mathrm H_4$	*Homo sapiens*	ERR324433	101	1,614,713,636	163,086,077,236	51	Build 38	3,209,286,105
$\mathrm H_5$	*Homo sapiens*	ERX069505	101	1,708,169,546	172,525,124,146	54	Build 38	3,209,286,105

**Fig. 1:**
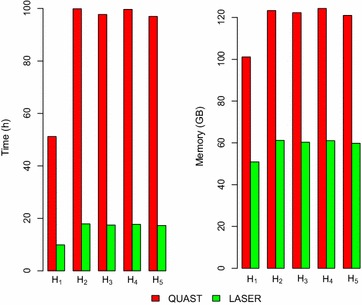
Comparison. Visual comparison of the time (*left plot*) and memory (*right plot*) between QUAST and LASER

Figure 1 gives the time and memory comparison between QUAST and LASER on the SOAPdenovo2 assemblies produced from the datasets in Table 1. LASER is 5.6 times faster than QUAST while using half the memory.

## Conclusions

We hope that the improvement in genome assembly evaluation provided by LASER will further boost the use of thorough quality evaluation. N50 is still used as the most important parameter. (N50 is the length *l* such that the sum of the lengths of all contigs of length *l* or more is at least half of the total length of all contigs.) An aggressive assembler will produce a high N50 but at the cost of many misassemblies, thus lowering the overall quality. Therefore, a combination of parameters, as provided by QUAST or LASER, gives a much better evaluation of the actual assembly quality.

## Availability and requirements

- Project name: LASER
- Project home page: http://www.csd.uwo.ca/~ilie/LASER/
- Operating system(s): UNIX, Linux, Mac OS X
- Programming language: C++, OpenMP
- License: see web page
- Any restrictions to use by non-academics: licence needed.
